# Investigating the utility of combining Φ29 whole genome amplification and highly multiplexed single nucleotide polymorphism BeadArray™ genotyping

**DOI:** 10.1186/1472-6750-4-15

**Published:** 2004-07-27

**Authors:** Rebecca Pask, Helen E Rance, Bryan J Barratt, Sarah Nutland, Deborah J Smyth, Meera Sebastian, Rebecca CJ Twells, Anne Smith, Alex C Lam, Luc J Smink, Neil M Walker, John A Todd

**Affiliations:** 1 Juvenile Diabetes Research Foundation/Wellcome Trust Diabetes and Inflammation Laboratory, Cambridge Institute for Medical Research, University of Cambridge, Wellcome Trust/MRC Building, Cambridge, CB2 2XY, UK

## Abstract

**Background:**

Sustainable DNA resources and reliable high-throughput genotyping methods are required for large-scale, long-term genetic association studies. In the genetic dissection of common disease it is now recognised that thousands of samples and hundreds of thousands of markers, mostly single nucleotide polymorphisms (SNPs), will have to be analysed. In order to achieve these aims, both an ability to boost quantities of archived DNA and to genotype at low costs are highly desirable. We have investigated Φ29 polymerase Multiple Displacement Amplification (MDA)-generated DNA product (MDA product), in combination with highly multiplexed BeadArray™ genotyping technology. As part of a large-scale BeadArray genotyping experiment we made a direct comparison of genotyping data generated from MDA product with that from genomic DNA (gDNA) templates.

**Results:**

Eighty-six MDA product and the corresponding 86 gDNA samples were genotyped at 345 SNPs and a concordance rate of 98.8% was achieved. The BeadArray sample exclusion rate, blind to sample type, was 10.5% for MDA product compared to 5.8% for gDNA.

**Conclusions:**

We conclude that the BeadArray technology successfully produces high quality genotyping data from MDA product. The combination of these technologies improves the feasibility and efficiency of mapping common disease susceptibility genes despite limited stocks of gDNA samples.

## Background

In order to locate the disease variants involved in complex common disease it is now generally accepted that very large sample numbers will be required [1-4]. Not only do the sample collections need to provide high quality gDNA, for the purpose of accurate genotyping, they also need to be sustainable. If, for example, one million SNPs were to be genotyped in a whole genome association scan and only 1 ng were required per SNP genotype, 1 mg of DNA would be required from each clinical sample. Given that the gDNA yield from a typical blood sample of 8 ml is approximately 200 μg, and that the typical yield from a mouth-swab is just 10 μg, there is clearly a short-fall in available quantities unless other means are employed to amplify the DNA resource. Moreover, many existing and effectively irreplaceable DNA sample collections, which have been used in previous studies and are now depleted, may consist of only nanogram quantities of gDNA.

At present, the gold standard method for generating gDNA from whole blood samples is through the process of immortalisation by transformation of the peripheral blood lymphocytes with Epstein-Barr Virus (EBV) [5]. Although this method of transfecting EBV creates an unlimited resource of gDNA, the procedure is costly, lengthy and not applicable to existing collections for which the gDNA has already been extracted. If there was a reliable method to enzymatically amplify the whole genome from nanogram-levels of gDNA and directly from clinical samples to microgram amounts then this would enable the use of archived gDNA in future studies, as well as providing an accelerated route to full use of newly collected clinical samples for high-throughput genotyping.

Molecular Staging, Inc. (MSI) (New Haven, CT, USA) have developed a method for whole genome amplification by Φ29 polymerase Multiple Displacement Amplification (MDA). It has been reported by the company that this method can reliably amplify the whole genome from gDNA, whole blood and other clinical samples [6-8]. Each DNA sample should give similar yields of product in all reactions with little dependency on the quantity of starting template [6,7]. Moreover, the MDA reaction should give complete coverage of the genome with little regional bias [6], which is critical when the product is to be used for high-throughput SNP genotyping. We set up a series of experiments with MSI in order to validate their claims that MDA product from gDNA is a viable alternative template to un-manipulated gDNA in SNP genotyping.

Recent studies have been conducted using MDA product from Amersham [9,10], and report the high level of accuracy achieved when these products are genotyped using TaqMan or multiplex, four-colour fluorescent minisequencing with six and 45 SNPs, respectively. However, without DNA resource limitations, a genotyping bottleneck exists mostly as a result of time- and assay set-up costs and hence, in order to achieve large-scale genotyping, highly multiplexed assays are required. In such multiplexed assays, there is greater potential for erosion of genotyping quality, due to reduced substrate integrity. The validation of the use of amplified DNA resources with such highly multiplexed methods is, therefore, essential.

The BeadArray genotyping platform of Illumina™ Inc. (San Diego, CA, USA) offers a high-throughput, highly multiplexed and highly automated genotyping service facility [11]. The BeadArray platform is highly miniaturised, using fibre optic bundles as a substrate for a high-density microarray [12]. It is the combination of this miniaturisation with an ability to multiplex up to 1,536 SNP assays [11] that makes BeadArray an attractive potential solution to the genotyping bottleneck. A recent study by Barker and colleagues, with 2,320 SNPs and five samples, found 99.86% concordance between MSI MDA product and gDNA [13]. However, since only five samples were studied it was not possible to evaluate accurately the efficacy of BeadArray on MDA product template, including estimation of sample exclusion and failure rates. In the present report we have, therefore, studied 86 MDA product samples and 384 SNPs using BeadArray, allowing comparison with the single-plex methods TaqMan^® ^(Assays-by-Design^SM^, Applied Biosystems, Foster City, CA, USA) and Invader^® ^(Third Wave Technologies, Inc., Madison, WI, USA) with gDNA.

## Results

### MDA yield

We selected and sent to MSI 20 ng of 88 gDNA samples for amplification, from which an average of 200 μg of MDA product was yielded in 100 μl reactions. The yields ranged from 85 μg to 280 μg, with 61% of samples yielding between 100 μg and 200 μg. The *HLA-DRB1 *genotype of each MDA sample was entirely concordant with the corresponding gDNA template, verifying the identity of each MDA sample and ruling out the possibility of contamination.

When 100 ng of 448 gDNA samples were amplified using reagents supplied by MSI in kit form and the amplification carried out in-house, an average of 155 μg of MDA product was yielded in 150 μl reactions. The yields ranged from 31 μg to 260 μg, with 80% of samples yielding between 100 μg and 200 μg.

### Compatibility of MDA product with TaqMan and Invader

**Figure 1:**
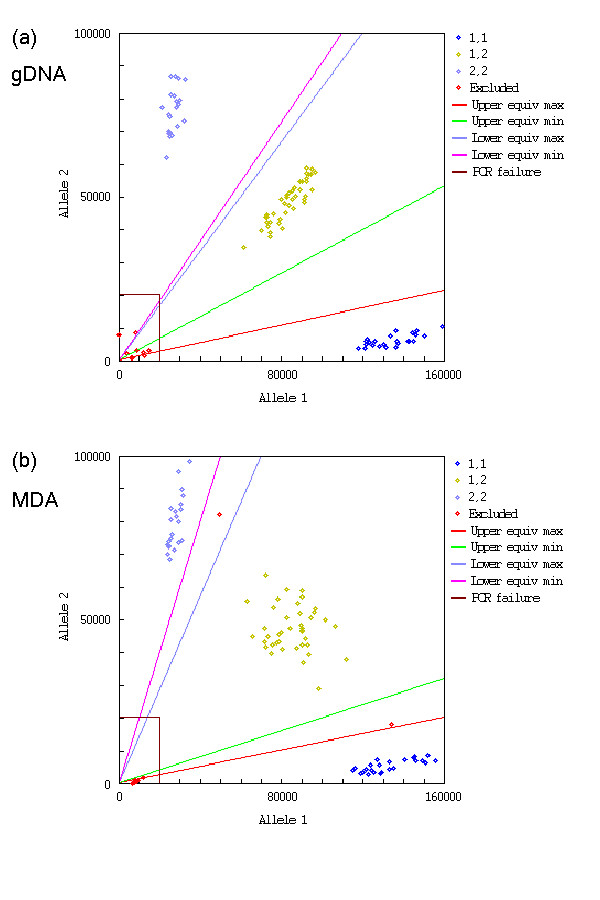
TaqMan fluorescence data plotted to compare the performance of MDA product with gDNA. This is a typical example of the clustering and scoring of TaqMan data for a common SNP in the evaluation of MDA product as a template for TaqMan genotyping. (a) End-point data from the 88 gDNA samples used in this evaluation and (b) the corresponding 88 MDA samples. In this example there are eight apparent failures for MDA, which are, in fact, empty wells. No genotypes were rejected for gDNA, and two genotypes were rejected for the MDA products due to poor clustering.

Of 88 MDA products and their corresponding gDNAs tested at 95 SNPs using the TaqMan method of genotyping there were no samples that consistently failed to produce any data. This confirmed that, for all samples, amplification had been sufficiently successful for the TaqMan chemistry to perform at most SNPs. Genotype concordance rates between MDA product and gDNA and genotype failure rates are given in Table 1. These results demonstrate that the use of MDA product as a template for the TaqMan assay produces accurate data comparable to that from gDNA. We observed that, for the majority of TaqMan assays, the clustering of data points was less distinct when MDA product was used as a template, compared to gDNA. Example data from an assay in which deterioration in clustering was observed are shown in Figure 1. For a single SNP of the 95 tested, the insulin gene (*INS*) -23*Hph*I (rs689), an allelic bias was observed in the MDA process, which resulted in the merging of the heterozygote cluster with the homozygote cluster of the major allele, making the correct assignments of genotype impossible, shown in Figure 2 [see additional file 1]. MDA product used as a template for the Invader genotyping method at this SNP produced similarly un-useable data. Using gDNA template at this SNP, however, both the TaqMan and Invader methods produced acceptable results, shown in Figure 2 [see additional file 1], indicating that the allelic bias was occurring at the MDA stage and not during subsequent genotyping. Interestingly, allelic bias has previously been observed at two other SNPs at *INS *in PCR reactions designed for the Pyrosequencing method (Pyrosequencing AB, Uppsala, Sweden) [14]. These results may indicate that *INS *may be situated in a sequence region that is predisposed to such allelic bias and the *INS *variable number of tandem repeats polymorphism, only 580 bp 5' to the -23*Hph*I SNP, is a candidate for such an effect.

For 13 additional SNPs for which Invader genotyping was performed, comparison of genotypes generated from MDA product with those from gDNA are shown in Table 1.

Two SNPs were genotyped by TaqMan on the 448 samples amplified using reagents supplied by MSI in kit form (and the amplification carried out in-house), and on the corresponding gDNAs. The gDNA samples used for amplification had been extracted from whole blood. Genotype concordance rates between MDA product and gDNA and genotype failure rates are given in Table 1.

### Validation of BeadArray genotyping technology with gDNA template

We commissioned Illumina to conduct a large-scale project using BeadArray genotyping technology involving 3,036 samples (2,950 gDNA samples and 86 MDA products) and 384 SNPs i.e. >1.1 million genotypes. In the first instance, 757 SNP sequences were sent to Illumina for *in silico *assay design. These SNPs were selected for their relevance to a range of ongoing projects in our laboratory, located at genes of strong functional candidacy and within regions of linkage to type 1 diabetes e.g. the putative *IDDM10 *locus on chromosome 10p14-11. All SNPs were validated, having been identified either from empirically confirmed SNPs in dbSNP or from our own re-sequencing efforts. Based on ranking from the *in silico *design criteria [15], 404 SNPs from 757 (53%) were suggested as most suitable for assay development, from which 384 were chosen. Thirty-nine of these failed to be converted into a viable assay (10.2%), leaving a total of 345 working assays.

As well as excluding SNPs that fail to produce robust genotypes, the Illumina protocol excludes samples that do not consistently perform. Of the total number of samples 10.5% were excluded and as a consequence very few data points were missing from the data set, resulting in an apparently low genotype failure rate (Table 2). Within the 2,781 successfully genotyped gDNA sample set, 26 were duplicate samples. Of these 52 samples at 345 SNPs, the genotypes of 23 duplicates did not match each other and 19 data points were missing, giving a discordance rate (error rate) of 0.26% (23 of 8,951 data points).

As our samples were family-based, a quality control check of misinheritance rates was possible using PedCheck [16]. Of the 345 SNPs, 20 displayed ≥ 10 misinheritances in the 742 families genotyped. For ten of these SNPs, TaqMan genotyping was attempted in the same samples in order to verify the results. It was possible to design TaqMan assays to only seven of the ten SNPs and, of these, only three produced interpretable data. At these three SNPs the numbers of misinheritances were 41, four and eight, respectively, by TaqMan, compared to 17, 22 and 14, respectively, by BeadArray. The number of SNPs with <10 or <5 misinheritances from the BeadArray experiment is shown in Table 3, along with our previous year's TaqMan results considering SNPs with allele frequencies >1%. The poor performance of both Illumina and TaqMan at the ten SNPs compared in detail, as well as the lab misinheritance rate for TaqMan (Table 3), indicates that the high misinheritance rates observed for some SNPs in the Illumina experiment is not a technology-specific failing.

Within the panel of 384 SNPs attempted by Illumina, 17 were controls for which we had already produced genotyping data by either TaqMan or Invader methods, enabling an evaluation of the BeadArray data for concordance. Two of these 17 control SNPs failed to be converted to a working assay, giving 15 SNPs and a maximum of 2,503 samples that were genotyped in common. Excluding failed duplicates noted above, comparison of BeadArray genotypes with existing data revealed a concordance rate of 99.6% (129 discordant in 34,219 comparisons), indicating the compatibility of the non-excluded gDNA samples with BeadArray, and the quality of existing data. Of the 15 control SNPs, 11 had been genotyped using TaqMan and four using Invader. The concordance rates for each platform were 99.7% using TaqMan (104 discordant in 25,203 comparisons) and 99.6% using Invader (25 discordant in 9,016 comparisons) when compared with BeadArray data, showing no significant difference between the two platforms.

### Compatibility of BeadArray with MDA product

Within the BeadArray experiment described above were 86 MDA products and their corresponding gDNA samples. These data were directly compared for sample failure rate and genotype failure rate as shown in Table 2. BeadArray genotype concordance rate between MDA product and gDNA are given in Table 1. These results provide evidence for the compatibility of the non-excluded MDA products with BeadArray technology. Evaluation of the Illumina's quality scores revealed no significant difference between the MDA and gDNA samples for any SNP (*t*-test *P-*value >0.05 for every SNP).

## Discussion

In this study we have evaluated the Φ29 polymerase MDA whole genome amplification method from MSI by assessing the compatibility of its product with the established TaqMan and Invader genotyping chemistries and with the highly multiplexed BeadArray genotyping platform. We have also evaluated Illumina's BeadArray genotyping platform for a large-scale experiment using gDNA.

At 95 SNPs, comparison of TaqMan genotypes generated from MDA product and gDNA templates revealed a very good concordance rate but a higher failure rate for MDA product compared to gDNA. This would need be estimated in a sample size larger than the current *n *= 88 in order to be confirmed. This result is comparable to the smaller study by Tranah *et al*. [9], in which six SNPs were genotyped by TaqMan on 172 samples, resulting in 100% concordance of pre- and post-MDA DNA. In the present study, the MDA product genotypes were slightly more difficult to assign, owing to more dispersed clusters. This was not observed by Lovmar *et al*. with fluorescent minisequencing on Amersham MDA products compared to gDNA [10]. One marker in our study, which may be unusually prone to allelic bias, was impossible to score using MDA product but was acceptable when using gDNA as a template (*INS *-23*Hph*I, rs689).

Compared to the yields indicated in Dean *et al. *[7], our average yield from in-house amplification using the reagents in kit form were in the order of five- to six-fold higher. This was probably due to differences in the two protocols: for example, our protocol used an increased reaction volume compared to the protocol used in Dean *et al. *[7]. Furthermore, the Dean *et al. *[7] protocol omitted the denaturation step, which is now standard practice. One other potential explanation for this variation is possible differences between laboratories in the quantitation of DNA using PicoGreen, the application of which requires a standard reference data set. We cannot at present fully resolve the differences in yields between studies but we can conclude that very large amounts of DNA are synthesized during the Φ29 reaction and that this is an excellent template for genotyping. MDA product should, therefore, be quantified and its concentration on completion of the MDA reaction not assumed to be consistent. Genotype failure rate, concordance rates with gDNA and the nature of genotype clustering showed similar patterns to service-generated MDA. However, a larger number of SNP markers would need to be genotyped on the MDA product using purchased kit reagents in order to verify these figures for in-house amplifications.

In the evaluation of MDA product in conjunction with BeadArray technology, the high concordance rate between genotypes obtained from MDA product and gDNA templates is encouraging. A concordance rate of 99.86% has been reported by Barker *et al*. using 2,320 SNPs and five samples [13]. However, as our study used 86 samples, we were able to observe differences in genotype failure rate between the different templates, not noted in the previous study [11]. As with the TaqMan evaluation, BeadArray had a higher genotype failure rate for MDA product compared to gDNA (0.2% for MDA versus 0.06% for gDNA). We did not find any evidence for allele drop-out with MDA compared to gDNA. BeadArray genotyping excluded more MDA samples than gDNA samples (10.5% for MDA versus 5.7% for gDNA) indicating that gDNA is a superior genotyping template for BeadArray technology. This 2-fold exclusion rate for MDA is consistent with the approximately 2 to 3-fold genotype failure rate of MDA typically observed with TaqMan and Invader, compared to gDNA (unpublished data).

The performance of MDA product is continuously being monitored in our laboratory. In a study blinded to all genotypers and database administrators, 288 family-based gDNA samples (prepared by the salting out method), were replaced with MDA product and left in continual use in our genotyping pipeline for 12 months. The change went undetected by all users. The failure rate for MDA was 3.34% for 15,921 genotypes, compared to 2.39% for 19,272 gDNA genotypes. Therefore, this improvement in the MDA performance for TaqMan is likely to be applicable to BeadArray, which improves the feasibility of mapping susceptibility loci in complex traits.

When using a highly multiplexed, highly automated genotyping platform, slight reductions in the quality of template material are likely to have a greater adverse effect on data than in scenarios in which markers are assessed individually and manual scoring is undertaken. Our results indicate that MDA is an adequate solution for the vast majority of SNP markers, even in this highly multiplexed allelic assay platform.

It is noted that 5.8% of markers that passed the Illumina acceptable scoring threshold were in fact showing high misinheritance rates in our family samples. This problem was at the same magnitude as TaqMan for individually genotyped markers. This highlights the importance of checking potential positive results with a second genotyping technology.

MDA should allow the continuation of genetic analysis on archived DNA in researchers' freezers worldwide, providing the very necessary increases in sample sizes so urgently required [1,2,17].

## Conclusions

The combination of BeadArray high throughput, multiplex genotyping and amplified DNA (MDA product) successfully produced high quality genotype data thereby improving the feasibility and efficiency of mapping common disease susceptibility genes despite limited stocks of gDNA samples.

## Methods

### MDA product preparation

For both the validation experiments (MDA product as a template for TaqMan and Invader genotyping) and for the combined experiment (MDA product as a template for BeadArray genotyping) the same MDA samples were tested. We sent to MSI 20 ng (5 μl at 4 ng/μl) of 88 gDNA samples for amplification, which was performed as a service according to the protocol for human gDNA with the omission of the denaturing step [7]. These gDNA samples had been extracted from cell pellets of EBV derived cell lines using a standard chloroform protocol that produces very high quality and stable gDNA [18]. The MDA product returned to us was quantified using PicoGreen dsDNA quantitation reagent (Molecular Probes Europe B.V., Leiden, the Netherlands).

In order to verify the identity of each MDA-produced sample, genotyping was performed at *HLA-DRB1 *and comparison made with data generated from the corresponding gDNA. *HLA-DRB1 *genotyping was performed using the Dynal Auto RELI™ SSO HLA-DRB Test system (Dynal^® ^Biotech, Wirrel, UK) for each gDNA sample and their MDA products. Although, these samples were amplified by MSI as a service, the reagents are also available from MSI in a kit form for amplification in-house. Following the amplification by MSI we have amplified 448 DNA samples by using the reagents in kit form and 100 ng (25 μl at 4 ng/μl) gDNA template in 100 μl reactions. In the interim, one major change to the MDA protocol had taken place, the inclusion of a denaturation of the DNA template prior to amplification. Previously no denaturation step took place. Two TaqMan markers were tested on these 448 samples and the genotype failure rate calculated.

### Evaluation of MDA product as a template for TaqMan and Invader genotyping

SNP TaqMan assays were carried out for allelic discrimination, 8 ng of DNA (2 μl at 4 ng/μl) used in a 5 μl total reaction volume. TaqMan genotypes from the 88 MDA samples, described above, were compared with TaqMan genotypes generated from their corresponding gDNAs, at 95 SNPs, with a broad range of allele frequencies. The Invader method was used to genotype 13 additional SNPs on the same samples. Comparison was made between data generated from both templates by the measurement of genotype failure and genotype concordance rates.

### Evaluation of BeadArray genotyping technology and its compatibility with MDA product

Of the 384 SNPs selected for genotyping 3,036 samples, 17 were control SNPs for which we had existing genotype data, generated by either TaqMan or Invader methods, with which comparison of genotype failure and genotype concordance rates were made. These 384 SNPs covered a broad range of allele frequencies.

Incorporated into this experiment was an assessment of suitability and compatibility of the BeadArray genotyping method with MDA product. This involved 86 of the 88 amplified samples, described above, for which genotyping was attempted at all 384 SNPS. Concordance between genotypes generated from MDA product and gDNA templates, together with the genotype and sample failure rates of each template type were measured.

In our laboratory we store genotyping data in a MySQL database on a Sun server. The volume of data expected from Illumina was the equivalent of 6 months' in-house genotyping. We separated phenotypic and pedigree information, which is associated with a sample, from genotype data, which is associated with a DNA plate and well position, with a link table to relate the two. Sample aliases are also supported, so that no recoding of identifiers is required, either to export or import Illumina data [19].

## Authors' contributions

RP participated in the design of the study, performed in-house genotyping experiments, and assisted in preparing the manuscript. HER prepared DNA and MDA amplified samples. BJB participated in the design of the study and assisted in the preparation of the manuscript. SN prepared DNA and MDA amplified samples. DS performed in-house genotyping experiments. MS prepared DNA and MDA amplified samples. RCJT participated in the design of the study and assisted in the preparation of the manuscript. AS participated in the design of the study. ACL and LJS performed bioinformatics. NMW managed the data and participated in its interpretation. JAT participated in the design of the study and assisted in the preparation of the manuscript. All authors read and approved the final version of the manuscript.

## Supplementary Material

Additional File 1 Figure 2 TaqMan and Invader fluorescence data plotted for the *INS *-23 HphI SNP. Both the MDA product plots could not be scored. All plots represent the same individual samples with gDNA plots containing 8 additional samples. Click here for file

## Figures and Tables

**Table 1:** Genotype concordance and failure rates for MDA compared with gDNA across genotyping platforms.

				Genotype Concordance Rate (%)	Genotype Failure Rate (%)	
Genotyping Platform	Site of MDA production	Number of SNPs	Number of non-excluded samples	gDNA and MDA product template	gDNA template	MDA product template

TaqMan	MSI	95	88	99.71	2.4	4.0
TaqMan	In-house	2	448	99.4	0.7	1.9
BeadArray	MSI	345	77	98.8	0.06	0.2
Invader	MSI	13	88	99.9	0.9	2.6

**Table 2:** BeadArray sample exclusion and genotype failure rates for MDA product compared with gDNA.

Template	Original number of samples	Number of excluded samples	Exclusion rate	Number of attempted genotypes	Number of failed genotypes	Genotype failure rate
gDNA	2,950	169	5.7	959,445	590	0.06
MDA product	86	9	10.5	26,565	52	0.2

**Table 3:** Comparison of misinheritance rates in families genotyped by BeadArray and TaqMan platforms using gDNA.

Genotyping Platform	Number of families tested	Number of SNPs tested	Number of SNPs with <10 misinheritances	Number of SNPs with <5 misinheritances
BeadArray	742	300	280 (93.3%)	248 (82.7%)
TaqMan	750	501	479 (95.6%)	409 (81.6%)
