# Supplementary figures and images for: Investigating the utility of combining Φ29 whole genome amplification and highly multiplexed single nucleotide polymorphism BeadArray™ genotyping
Source: BMC Biotechnol. 2004 Jul 27;4:15. doi: 10.1186/1472-6750-4-15 (PMC514612; doi:10.1186/1472-6750-4-15)

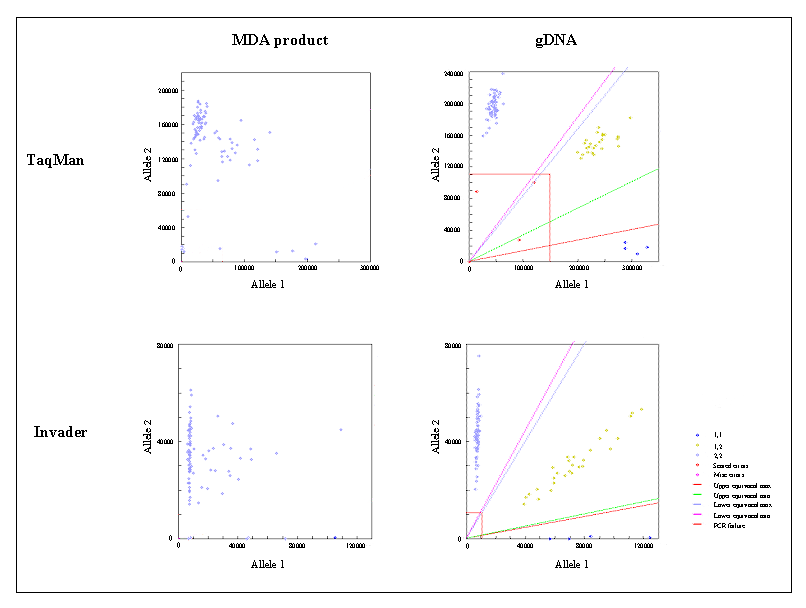

Supplement: Additional File 1 — Figure 2 TaqMan and Invader fluorescence data plotted for the INS -23 HphI SNP. Both the MDA product plots could not be scored. All plots represent the same individual samples with gDNA plots containing 8 additional samples. [file 1472-6750-4-15-S1.TIFF]
